# Long-term timing stabilization for pump–probe experiments at SACLA

**DOI:** 10.1107/S1600577524011974

**Published:** 2025-02-03

**Authors:** Tadashi Togashi, Shigeki Owada, Toshinori Yabuuchi, Makina Yabashi

**Affiliations:** a Japan Synchrotron Radiation Research Institute (JASRI) Sayo-cho Sayo-gun679-5198 Japan; b Riken SPring-8 Center, Sayo-cho, Sayo-gun679-5148, Japan; University of Malaga, Spain

**Keywords:** X-ray free-electron laser, time-resolved pump–probe experiment, timing stabilization

## Abstract

We have developed a timing control system to stabilize the long-term timing drift between X-ray free-electron laser (XFEL) and optical laser pulses using an out-of-loop balanced optical-microwave phase detector and an arrival-timing monitor for pump–probe experiments at the SPring-8 Ångstrom Compact free-electron LAser (SACLA). The timing jitter and drift between the XFEL and the optical laser pulses have been reduced to less than 50 fs (RMS) over ∼49 h. The performance of the timing stabilization system was investigated by measuring the correlation of the long-term simultaneous timing monitoring on two branches of BL3 over 8 h. A linear correlation was observed with an RMS error of 8.6 fs.

## Abstract 2

A timing control system to stabilize the long-term timing drift between X-ray free-electron laser (XFEL) and optical laser pulses has been developed by utilizing an out-of-loop balanced optical-microwave phase detector and an arrival-timing monitor. The timing jitter and drift between the XFEL and the optical laser pulses have been reduced to less than 50 fs (RMS) over ∼49 h.

## 1. Introduction

X-ray free-electron lasers (XFELs) (*e.g.* Emma *et al.*, 2010 ▸; Ishikawa *et al.*, 2012 ▸) have led to significant advancements across various fields, including biology, chemistry, materials science and physics. One of the most active areas of XFEL applications is the investigation of ultrafast dynamics in chemical reactions and phase transitions involving structural changes on a femtosecond time scale using pump–probe techniques with femtosecond optical lasers (*e.g.* Verma *et al.*, 2024 ▸; Suzuki *et al.*, 2023 ▸; Katayama *et al.*, 2023 ▸; Gruhl *et al.*, 2023 ▸). When optical lasers are synchronized with a reference signal provided by an XFEL machine via a standard phase-locking system, several sources of error can introduce sub-picosecond timing jitters between XFEL and optical laser pulses, which limits the time resolution in pump–probe experiments. To fully exploit the ultrashort pulse nature of XFELs, shot-to-shot diagnostics of the arrival timing between the XFEL and optical laser pulses are essential. At SPring-8 Ångstrom Compact free-electron LAser (SACLA) (Ishikawa *et al.*, 2012 ▸), sub-10 fs resolution has been achieved by employing a post-process sorting method with an arrival-timing monitor (ATM) (Sato *et al.*, 2015 ▸; Katayama *et al.*, 2016 ▸; Nakajima *et al.*, 2018 ▸; Owada *et al.*, 2018 ▸; Owada *et al.*, 2019 ▸).

However, a higher precision timing stabilization system is required to increase the number of events at specific delay timings. Femtosecond-scale timing stabilization systems have been developed at facilities such as PAL-XFEL (Kang *et al.*, 2017 ▸), the European XFEL (Sato *et al.*, 2020 ▸) and other XFEL facilities. At SACLA, we developed a direct synchronization system using a balanced optical-microwave phase detector (BOMPD) operating at 5.7 GHz RF signals from the accelerator as an *in-loop* detector (Togashi *et al.*, 2020 ▸). While this system allowed for the reduction of a short-term timing jitter to approximately 50 fs (RMS), a long-term timing drift of 0.5 ps remained after a day due to variations possibly in optical path length, room temperature, humidity and other factors. In this paper, we present the development of a timing stabilization system utilizing an *out-of-loop* BOMPD and an ATM, which have been installed as timing monitors for the synchronization system, to compensate for the long-term timing drift. First, details of the laser synchronization system and the monitor performance of the out-of-loop BOMPD and the ATM are described in Section 2. Next, the timing drift controls utilizing the out-of-loop BOMPD and the ATM are presented in Sections 3 and 4, respectively.

## 2. Laser synchronization system

**Figure 1:**
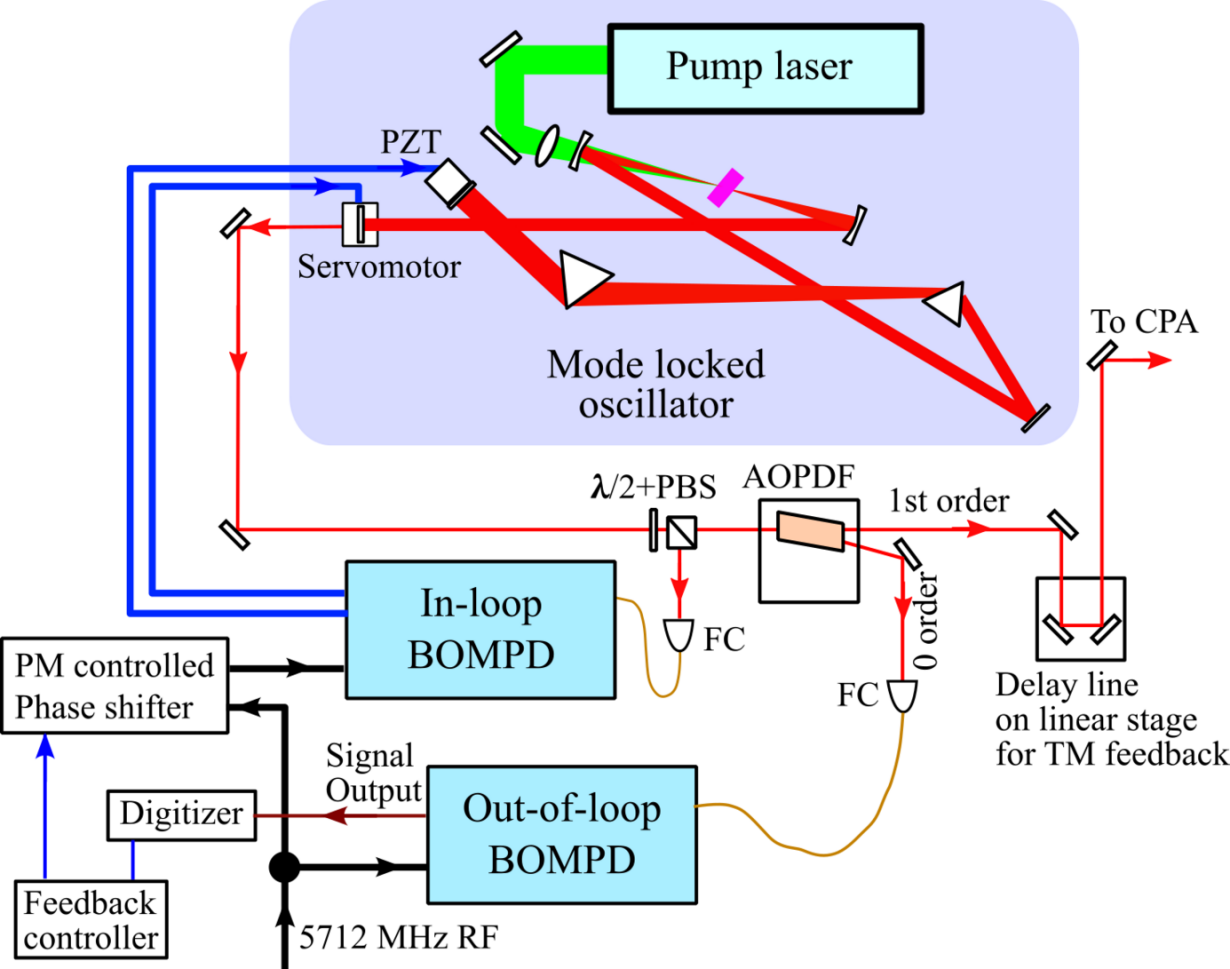
Schematic diagram of the synchronization system using a balanced optical-microwave phase detector (BOMPD). The phase-error signal between the pulse train generated by the mode-locked oscillator (79.3 MHz) and the reference RF (5.7 GHz) is measured by the in-loop BOMPD and applied to the oscillator cavity length. Long-term drift is monitored by the out-of-loop BOMPD and compensated using the pulse-motor-controlled RF phase shifter inserted before the RF input to the in-loop BOMPD. The optical delay line, installed before the regenerative amplifier in the chirped pulse amplification system, is used to adjust the relative timing between the XFEL and optical laser pulses. λ/2+PBS: half-wave plate and a polarization beam splitter; FC: fiber coupler; AOPDF: acousto-optic programable dispersive filter.

**Figure 2:**
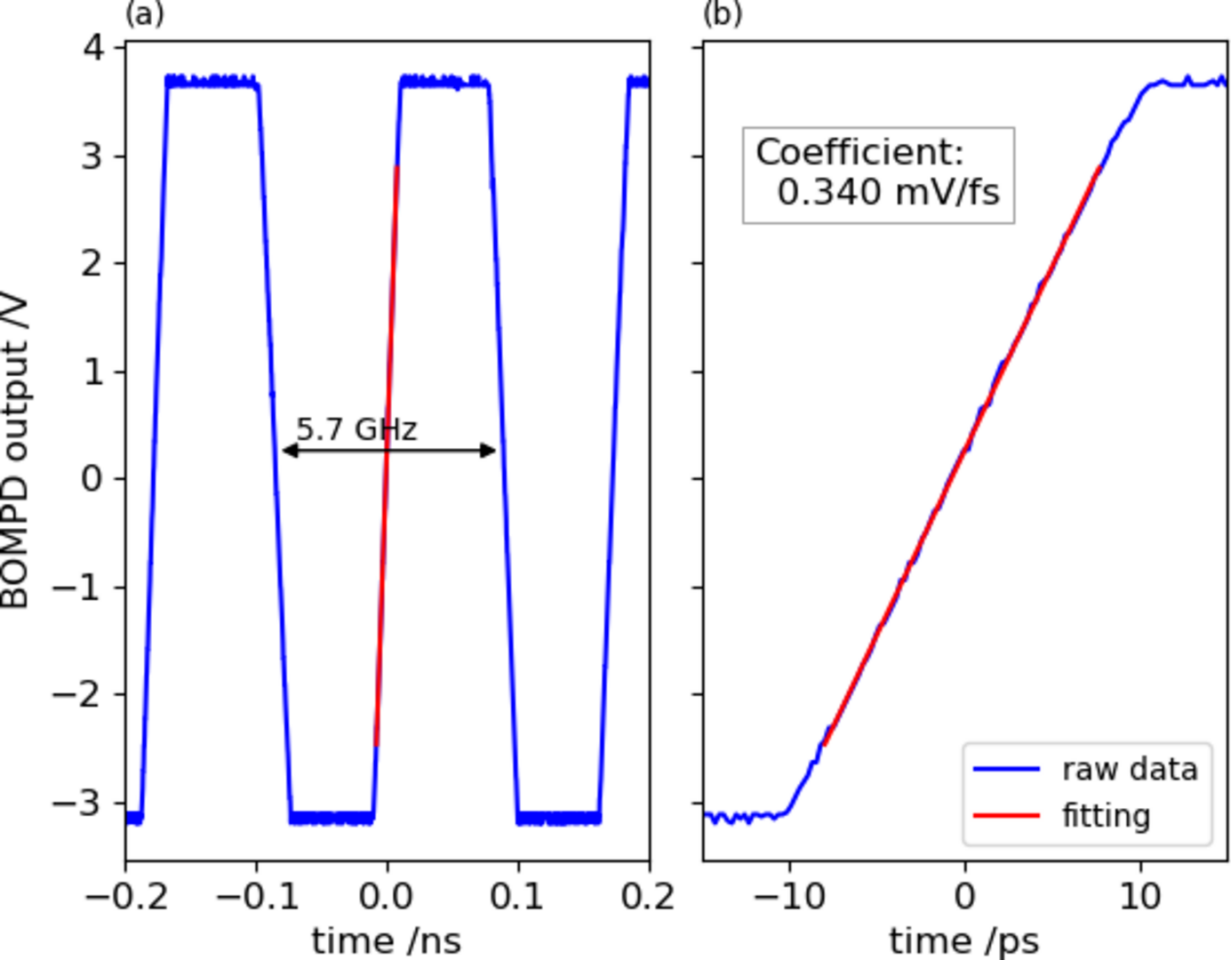
Out-of-loop BOMPD output trace (blue trace) without synchronization. The horizontal axis is calibrated to the real-time scale of the BOMPD response with the ratio of the beat frequency over the RF frequency (5.7 GHz). The time expansion around 0 of (*a*) is shown in (*b*). The sensitivity is defined as 0.34 mV fs^−1^ from the zero-crossing slope (red trace).

**Figure 3:**
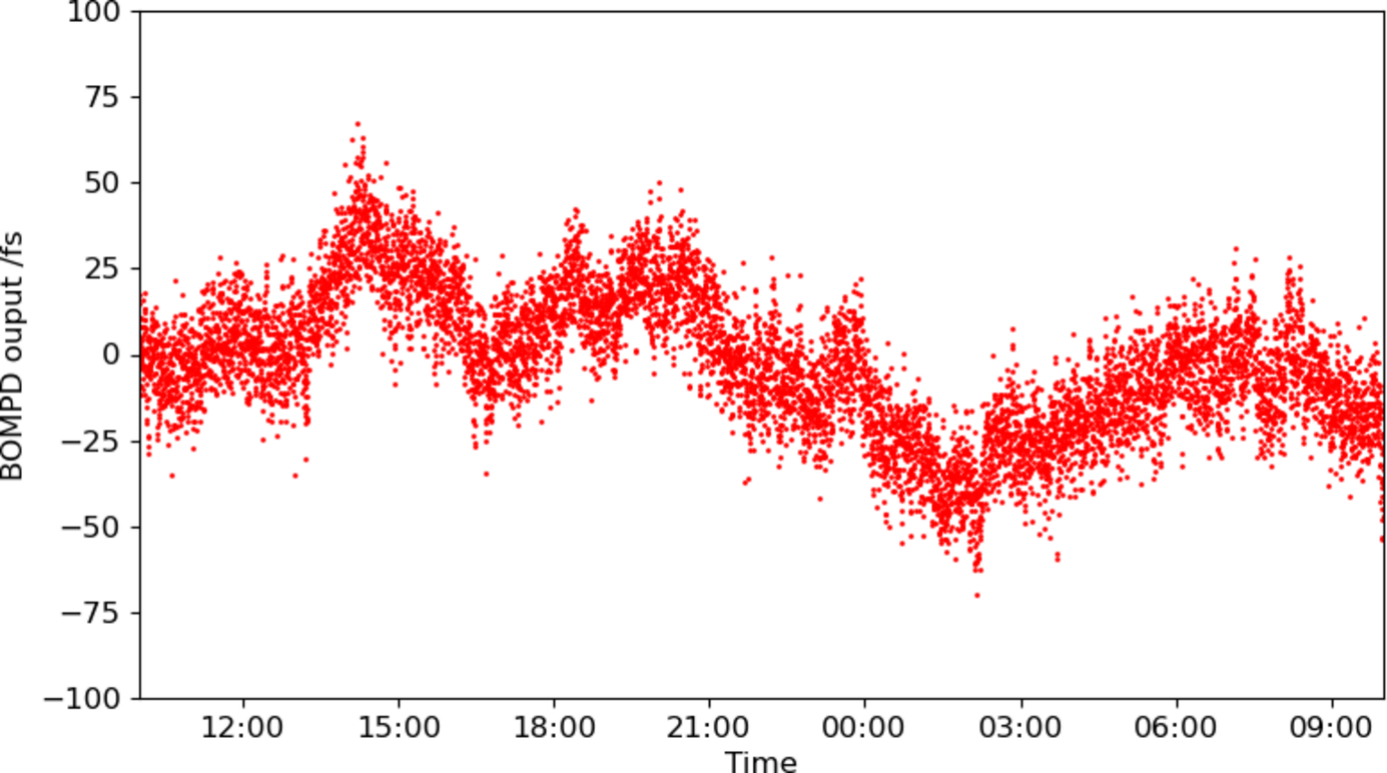
Out-of-loop BOMPD output trend in 24 h without drift control. The horizontal time range is the same as that in Fig. 5 ▸.

The synchronized optical laser system, which is based on a chirped pulse amplification (CPA) of a Ti:sapphire laser, consists of a mode-locked oscillator, a pulse stretcher, a regenerative amplifier, a multi-pass amplifier and pulse compressors. This laser system provides output pulses of 12 mJ with about 40 fs pulse duration for user experiments. Precise synchronization to XFEL pulses is achieved by controlling the cavity length of the mode-locked oscillator, which generates a pulse train at 79.3 MHz, using the 5.7 GHz RF signal for the accelerator operation as illustrated in Fig. 1 ▸. The timing between the pulse train from the oscillator and the RF signal is detected by BOMPDs as a phase-error-dependent intensity imbalance between two outputs from a Sagnac-loop interferometer (Kim *et al.*, 2004 ▸; Jung & Kim, 2012 ▸; Peng *et al.*, 2014 ▸). The repetition rate of the oscillator is locked by applying the phase error signal from the in-loop BOMPD (BOMPD-8-SD, Cycle GmbH) to the linear actuator of the cavity mirror in the mode-locked oscillator. Generally, a feedback control system suppresses all noise sources with frequencies higher than the locking bandwidth, making it insensitive to frequencies lower than the loop interval. Therefore, an out-of-loop BOMPD (BOMPD-8-MD, Cycle GmbH) has been installed to monitor the long-term relative phase error, *i.e.* the timing between the mode-locked oscillator and the RF signal. The 5.7 GHz RF signals used for the in-loop and the out-of-loop BOMPDs as reference are divided just before the BOMPDs’ inputs and connected with short cables to avoid the influence of electrical noise and thermal extension. About 10% of the oscillator output, divided by a polarization beam splitter (PBS) and a half-wave plate at the oscillator exit, is required as the in-loop BOMPD input for a stable feedback loop. The zero-order output from an acoustic-optic programable dispersive filter (AOPDF) (Dazzler, FASTLITE) (Verluise *et al.*, 2000 ▸), which can compensate for and optimize dispersions in the optical path, is used for the out-of-loop BOMPD because the remaining beam split by the PBS has the almost minimum pulse energy required as a seeder of the regenerative amplifier. We calibrated the out-of-loop BOMPD sensitivity as 0.34 mV fs^−1^ from the BOMPD output trace without the locking. The beat frequency *f*_beat_ of the output trace is defined when the oscillator is unlocked as
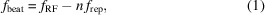
where *f*_RF_ is the frequency of the RF source (5.7 GHz), *f*_rep_ is the repetition rate of the oscillator (79.3 MHz), and *n* is a maximum natural number so that the beat frequency *f*_beat_ is the remainder of *f*_RF_ divided by *f*_rep_. The real-time scale of the BOMPD response *t*_BOMPD_ is obtained by multiplying the recorded time *t*_OSC_ on the oscilloscope measuring the output trace with the ratio *f*_beat_/*f*_RF_ (Kim *et al.*, 2007 ▸),
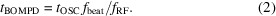
The linear slope at the zero crossing in the output trace gives the sensitivity as shown in Fig. 2 ▸. We analyzed the phase error at 47 fs from the phase noise spectral density of the out-of-loop BOMPD output locked by the in-loop BOMPD, which is measured by a phase noise analyzer (FSWP8, Rohde & Schwarz GmbH & Co. KG) (Togashi *et al.*, 2020 ▸). However, the long-term drift is about 0.1 ps in 24 h as shown in Fig. 3 ▸.

**Figure 4:**
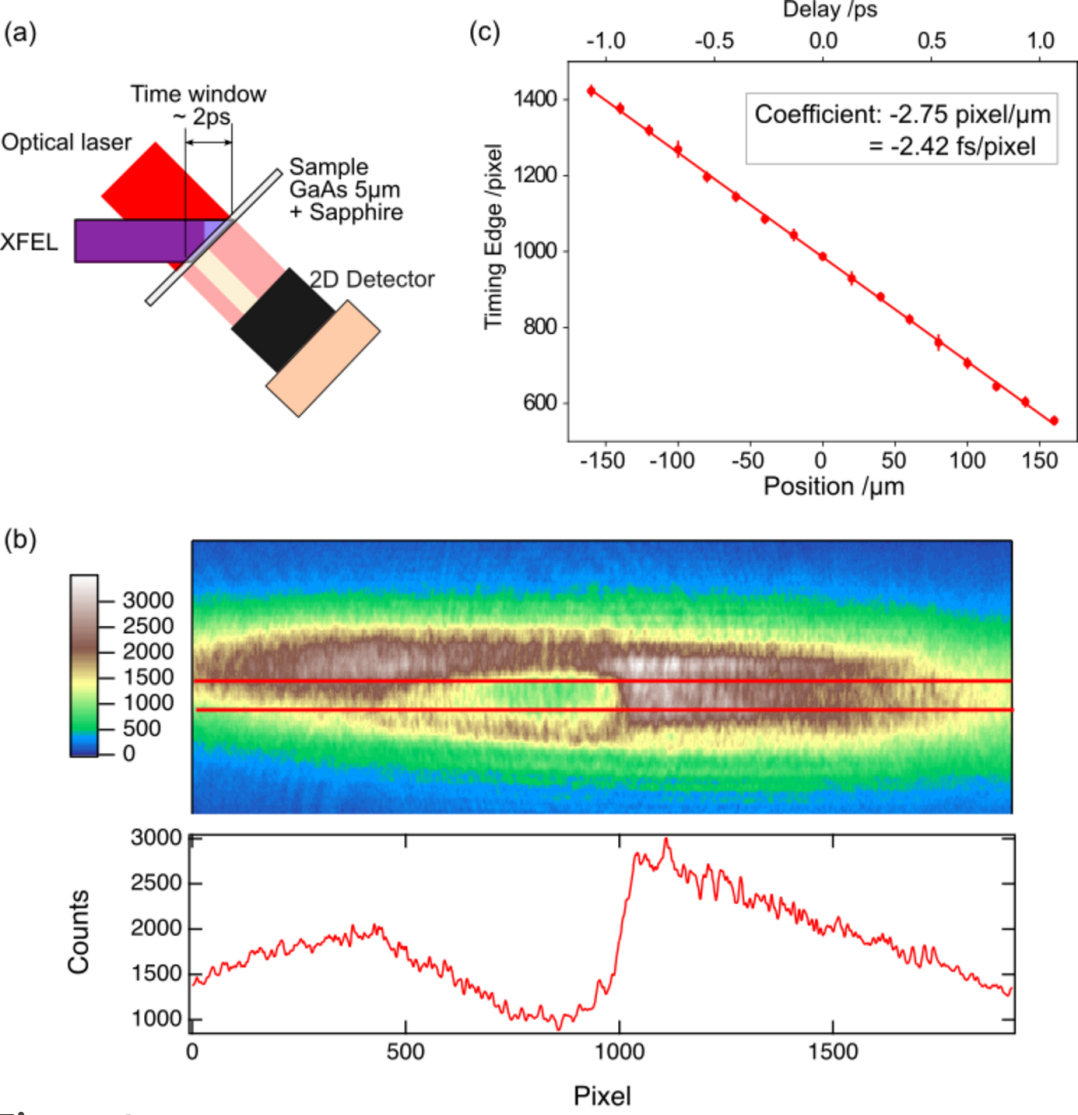
(*a*) Schematic of the ATM setup. (*b*) Camera image of the ATM (top) and vertical projection trace in the irradiated area by the XFEL (indicated by red lines) (bottom). (*c*) Timing edge in pixels as a function of the stage position of the delay line. The conversion coefficient is calibrated as 2.42 fs pixel^−1^ based on the linear fit.

To investigate and compensate the timing fluctuation between the XFEL and the optical laser pulses, we have developed and have been operating an ATM (Sato *et al.*, 2015 ▸; Katayama *et al.*, 2016 ▸; Nakajima *et al.*, 2018 ▸). The ATM is based on a spatial decoding method utilizing a transient optical transmittance change of gallium arsenide (GaAs) when irradiated by intense X-ray pulses. The data set of the pump–probe experiments can be sorted according to the timing data simultaneously measured by the ATM, allowing for post-processing correction of errors due to the timing fluctuation. Fig. 4 ▸(*a*) shows a schematic design of the ATM at BL3. The horizontal incident angle is set at 45° to the target for spatial decoding. The optical laser beam propagates along the surface normal of the target (forming the 45° crossing angle toward the XFEL beam) and probes the area irradiated by the XFEL beam. The ATM is installed on the −1st-order branch split by a one-dimensional transmission grating in EH1. The 0th-order branch is utilized for user experiments in the downstream experimental hutches (EH2 or EH4a), while the 1st-order branch is used for the single-shot spectrometer in EH1 (Katayama *et al.*, 2016 ▸). Images of the transmitted laser beam are captured by a long-working-distance microscope and a charge-coupled-device (CCD) camera (OPAL-2000, Adimec). Fig. 4 ▸(*b*) shows a typical camera image from the ATM at BL3, along with the projection trace of the area irradiated by the XFEL beam [between the red lines in Fig. 4 ▸(*b*, top)]. The pulse front of the XFEL beam creates a sharp edge in the projection trace. The spatial decoding method allows this edge position to be converted into the arrival time of the optical laser pulse with respect to the XFEL pulse. The conversion coefficient is calibrated as 2.42 fs pixel^−1^ by scanning the delay line, as shown in Fig. 4 ▸(*c*). We have also developed ATM software to analyze these sequences and record the timing data for user experiments (Nakajima *et al.*, 2018 ▸).

**Figure 5:**
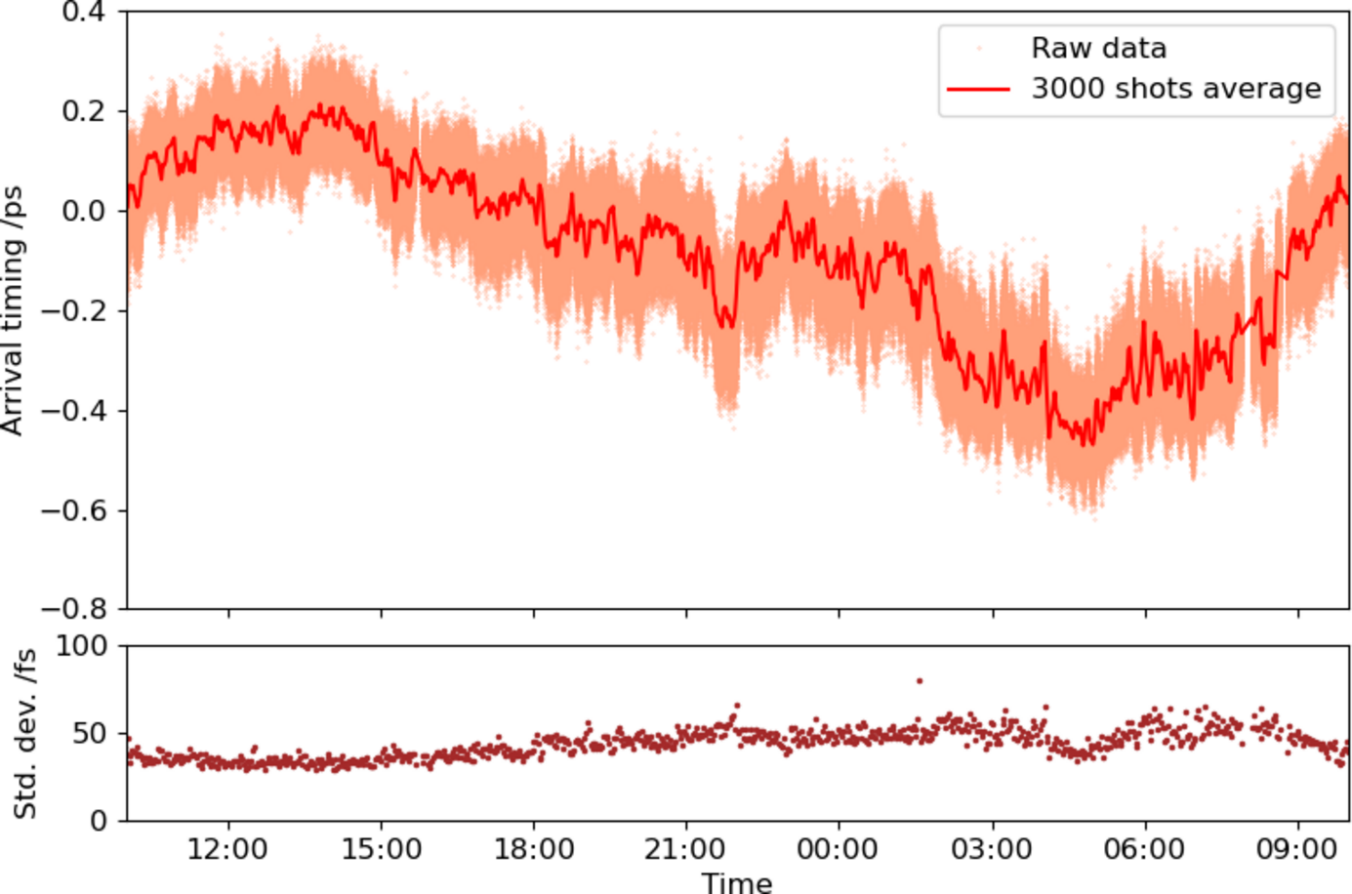
Arrival timing trend measured by the ATM over ∼24 h (2619007 shots in 30 Hz) without drift control. The orange dots are raw data. The red and brown traces are average and standard deviation in 3000 shots, respectively. The time range is the same as that in Fig. 3 ▸.

Fig. 5 ▸ shows the arrival timing trend over ∼24 h, which the ATM measured simultaneously with the trend of the out-of-loop BOMPD shown in Fig. 3 ▸. While the synchronization system using the in-loop BOMPD has reduced timing fluctuations to 50 fs (RMS), a long-term timing drift of ∼0.5 ps per day remains. This indicates that the timing drift between the XFEL and the optical laser pulses would be due not only to the synchronization system as measured by the out-of-loop BOMPD (shown in Fig. 3 ▸) but also to other factors.

We have implemented the out-of-loop BOMPD and the ATM systems for the available monitors of the timing drift control, as explained in the previous paragraphs. The out-of-loop BOMPD continuously operates by referring to the standard 5.7 GHz RF signal, which governs all accelerator components at SACLA and can detect the changes within the propagation path for the timing signal. However, this system alone is insufficient for addressing changes in the CPA system and optical transport. In contrast, the ATM can directly and precisely detect the timing of the optical laser pulse, referenced to the XFEL pulse, although limited during the XFEL operation. The timing control using the out-of-loop BOMPD is effective for coarse adjustments owing to its wide linearity range of ±8 ps (Fig. 2 ▸). Meanwhile, the ATM system, with a range of ±1 ps [Fig. 4 ▸(*c*)], is suitable for fine-tuning. In this study, we developed timing drift control systems utilizing both the out-of-loop BOMPD and the ATM, as detailed in Sections 3 and 4, respectively.

## 3. Timing control with the out-of-loop BOMPD

**Figure 6:**
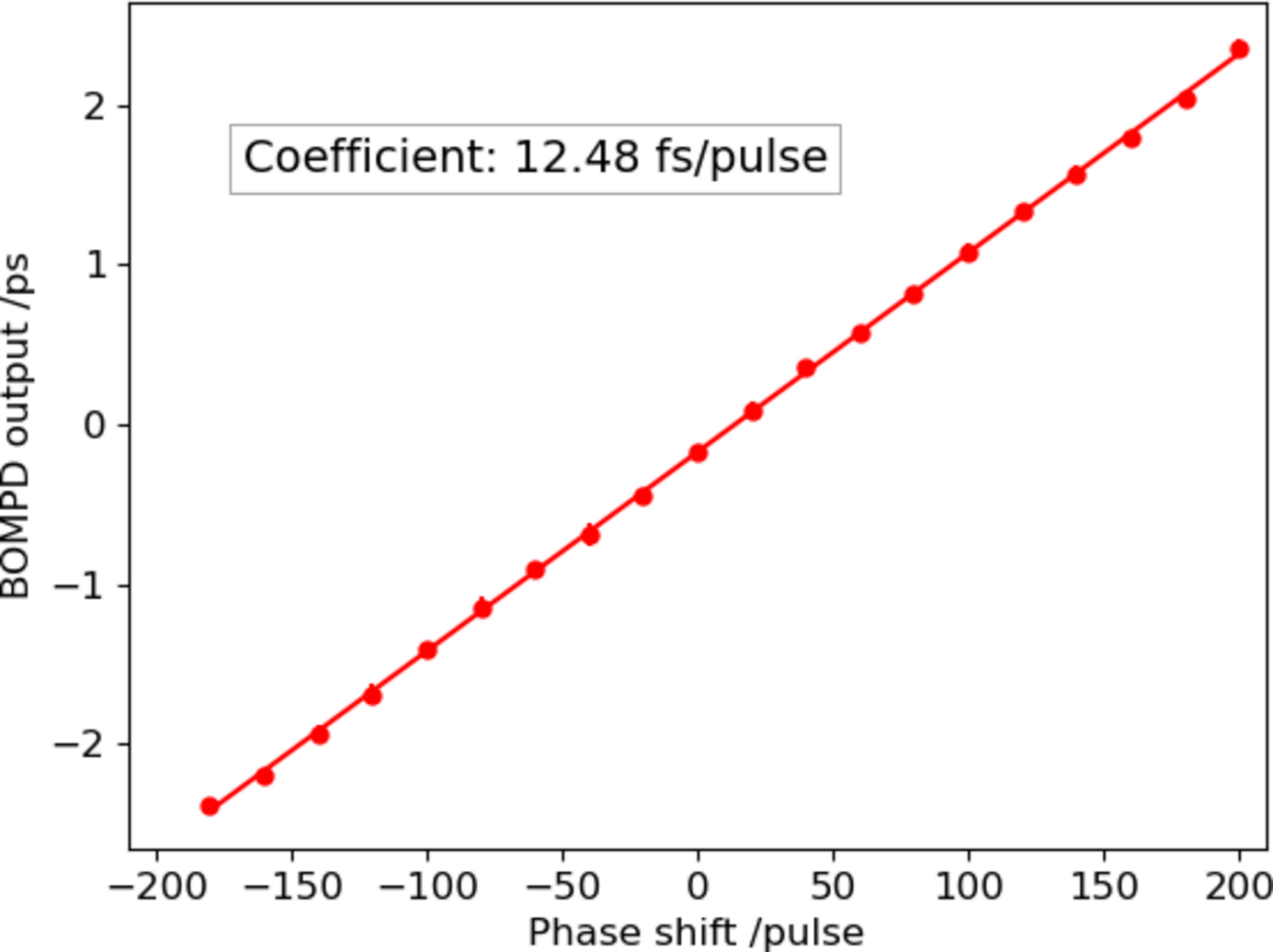
Out-of-loop BOMPD output as a function of the position of the pulse motor-controlled phase shifter. The coefficient is defined as 12.48 fs pulse^−1^ by the linear fit.

**Figure 7:**
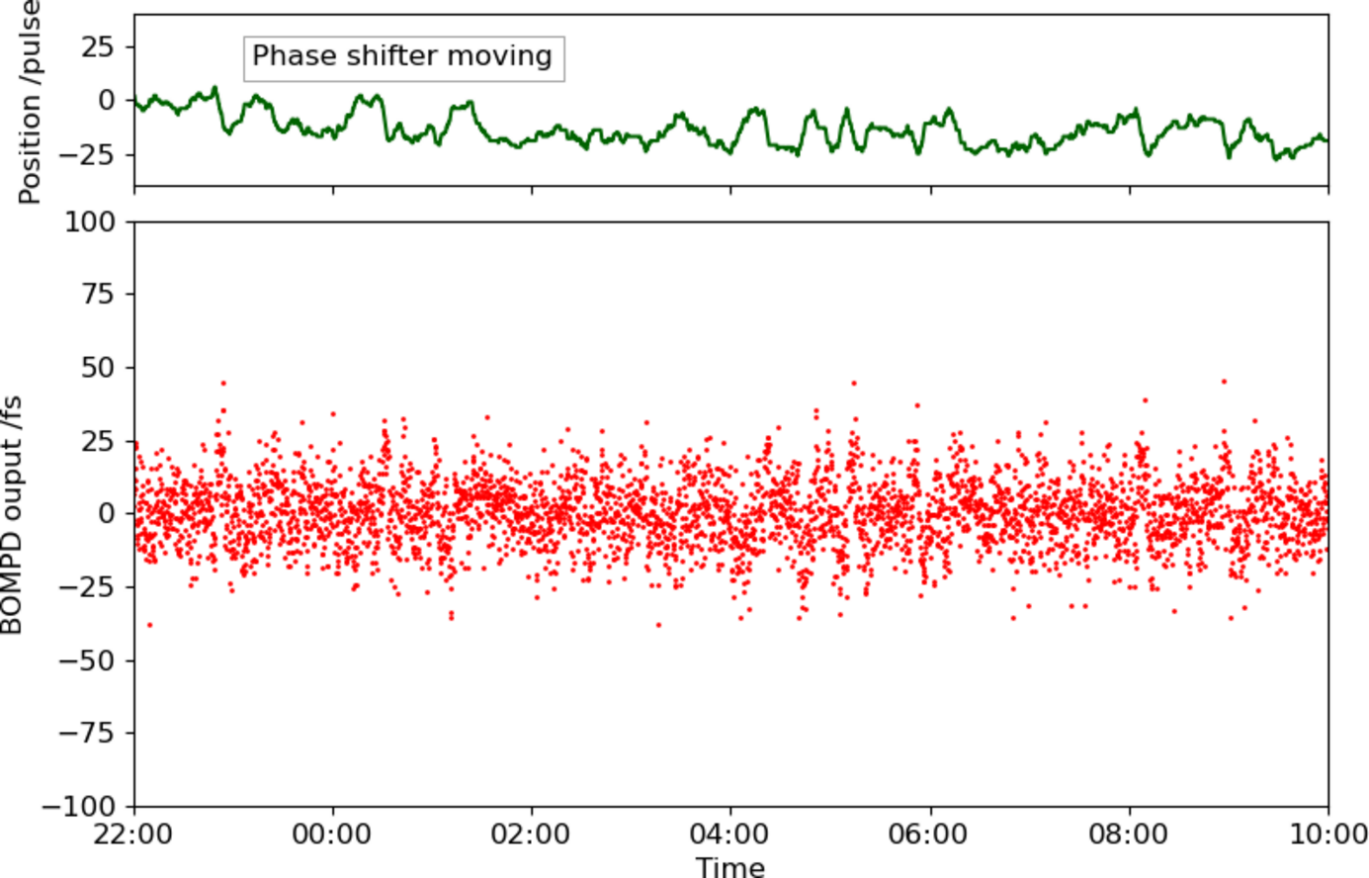
Trends of the out-of-loop BOMPD output and the phase shifter position controlling the timing drift in 12 h. The time range is the same as that in Fig. 8 ▸.

**Figure 8:**
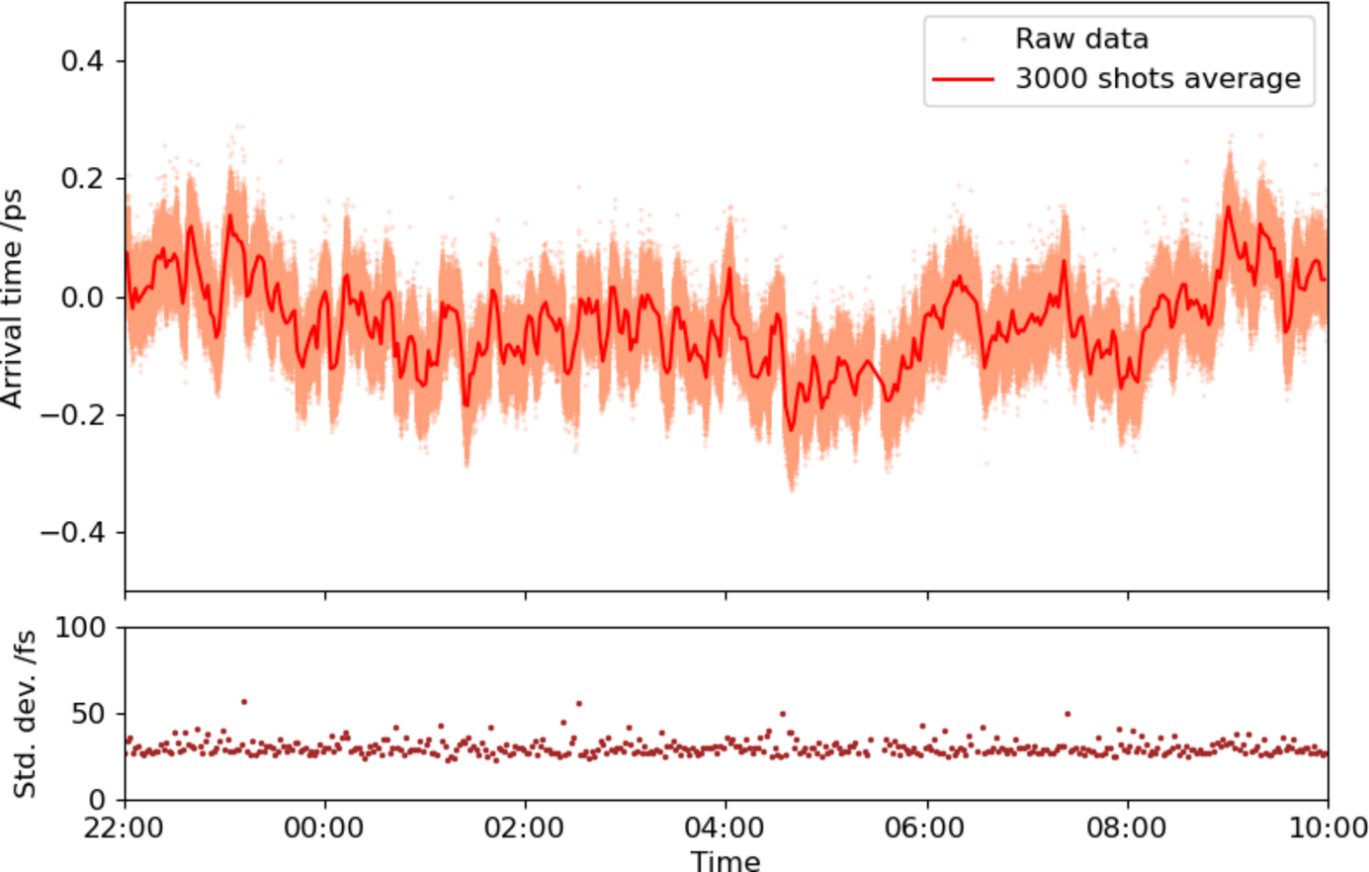
Arrival timing trend measured by the ATM controlling the long-term timing drift with the out-of-loop BOMPD in 12 h (1403563 shots in 30 Hz). The red and brown traces are the average and standard deviation in 3000 shots, respectively. The time range is the same as in Fig. 7 ▸.

To control the optical laser timing relative to the RF signal, we inserted the pulse motor controlled RF phase shifter (SSX-0058, WAKA Manufacturing Co.) before the RF input of the in-loop BOMPD (Fig. 1 ▸). We calibrated the phase shifter with the out-of-loop BOMPD before the timing control operation. Fig. 6 ▸ shows the out-of-loop BOMPD output as a function of the phase shifter position. The coefficient of the phase shifter is 12.48 fs pulse^−1^. To operate the timing control system, the out-of-loop BOMPD output is acquired by a digitizer (DMM6500, Tektronix) at the laser system’s repetition rate of 60 Hz. The feedback controller averages 100 samples in the digitizer memory and adjusts the phase shifter to maintain the out-of-loop BOMPD output at zero, with a feedback interval of 10 s. Fig. 7 ▸ shows the trends of the out-of-loop BOMPD output and the phase shifter position during the feedback operation of 12 h. On the other hand, Fig. 8 ▸ shows the trends of the arrival timing, which the ATM simultaneously measured with the trend of the out-of-loop BOMPD shown in Fig. 7 ▸. These measurements showed a long-term timing drift of ∼0.2 ps, which means a timing improvement compared with one without the timing control shown in Fig. 5 ▸. The remaining drift may be caused by optical path changes in the multiple amplifiers and transport after the oscillator, related to temperature, humidity and airflow.

## 4. Timing control with the ATM

**Figure 9:**
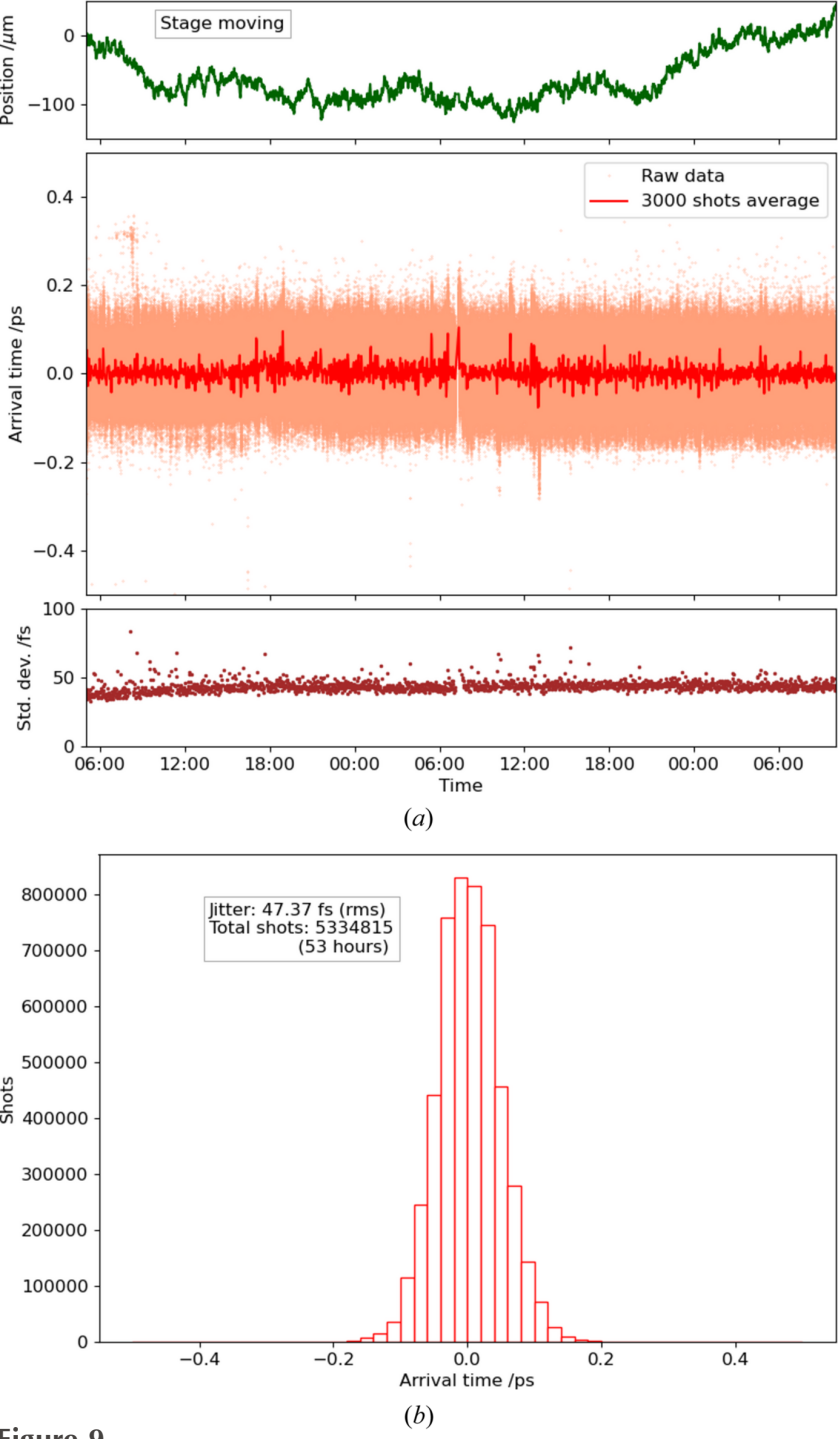
(*a*) Trends of the arrival timing measured by the ATM and (*b*) histogram, controlling the timing drift over 49 h (5334815 shots in 30 Hz). In (*a*), the orange dots are raw data, while the red and brown traces are average and standard deviation in 3000 shots, respectively. The green trace in (*a*) is the stage position of the delay line.

For further improvements in the timing stabilization, we developed a direct control system for adjusting the relative timing between the XFEL and the optical laser pulses by applying the ATM data to an optical delay line. The optical delay line on a linear stage (V-522.1AA, Physik Instrumente GmbH) was installed before the first regenerative amplifier in the CPA system because the output beam configuration is mostly dominated by the cavity mode of the regenerative amplifier and tolerant to the position and the direction of the input seeding beam (Fig. 1 ▸). We also confirmed that the beam position at the sample remained stable when the delay line moved in the full range of the linear stage. The control system adjusts the optical delay line with a feedback interval of 10 s based on the average of the latest 100 shots’ data point recorded by the ATM in the user database of the SACLA data aquisition system (Nakajima *et al.*, 2018 ▸). Fig. 9 ▸(*a*) indicates the trends of the timing data and the stage position of the delay line during the feedback operation over 49 h. We succeeded in reducing the long-term timing fluctuation to 50 fs, as given in Fig. 9 ▸(*b*) which shows a histogram of the timing data of Fig. 9 ▸(*a*). Because this long-term timing fluctuation is at a similar level to the short-term timing jitter, this method significantly reduced the long-term drift.

**Figure 10:**
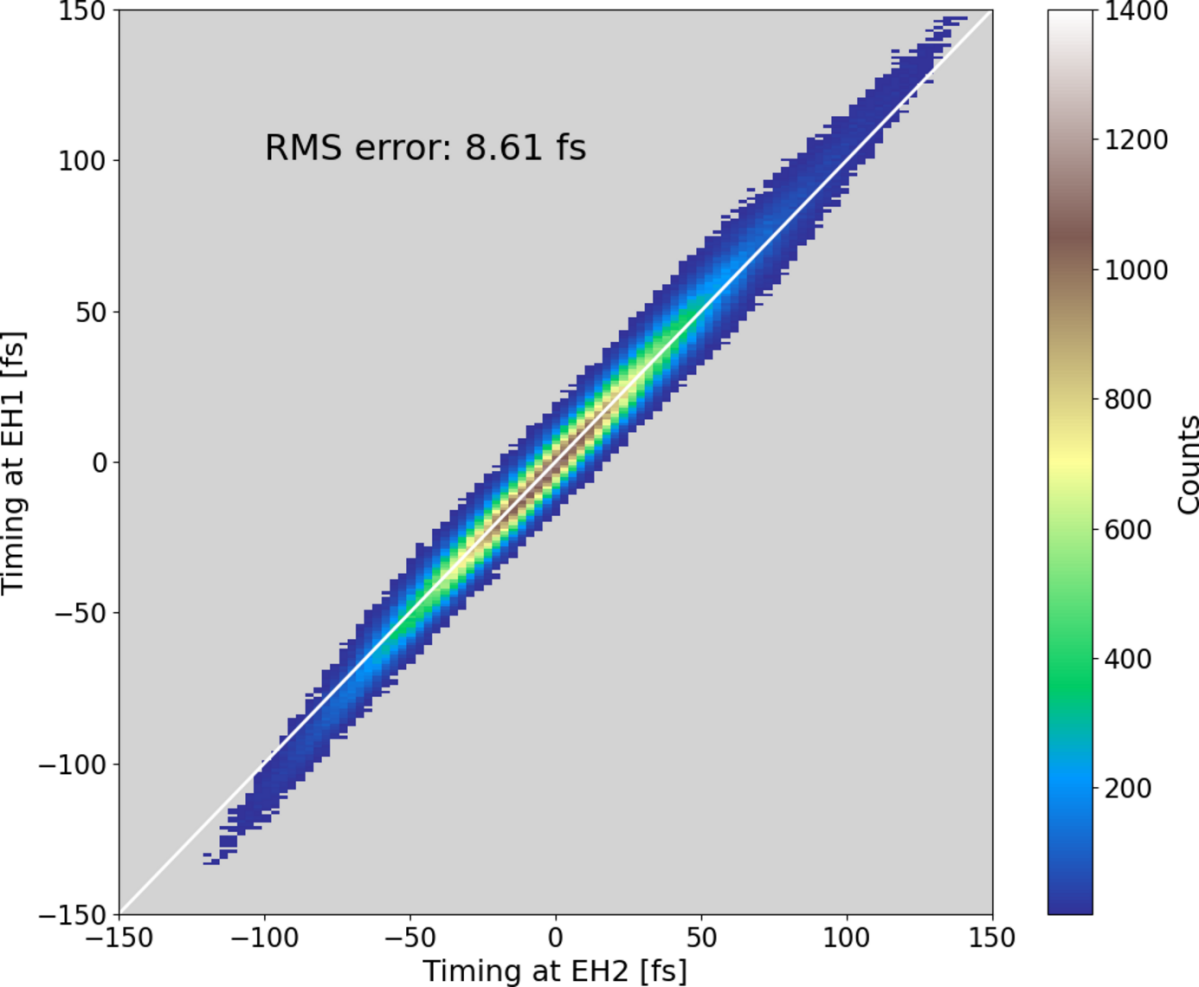
Two-dimensional histogram of the correlation between the arrival timings of the −1st-order branch in EH1 and the 0th-order branch in EH2 in ∼8 h (9844791 shots with 30 Hz).

To evaluate the performance of the temporal stabilization systems, we simultaneously conducted timing measurements between the XFEL and the optical laser pulses at two places, *i.e.* at the ATM in EH1 and at the sample position in EH2, and investigated the correlation. The XFEL beam was split into two branches, −1st-order for EH1 and 0th-order for EH2, with a grating beam splitter (Katayama *et al.*, 2016 ▸). Fig. 10 ▸ shows the correlation plot with a two-dimensional histogram in 8 h. The result is fit with a linear function shown as the white line in Fig. 10 ▸. The coefficient of the timing measurement on the 0th-order branch is determined as 3.25 fs pixel^−1^ from the correlation. The RMS error from the line is 8.6 fs, which may have originated from mechanical vibrations of the optics and the samples, optical pointing fluctuations, and imperfection of the sample flatness. This timing fluctuation of lower than 10 fs enables the temporal accuracy of the pump–probe experiments to improve after re-sorting the data using the ATM, although it is mainly limited by the pulse duration of the optical laser.

## 5. Conclusion

We have developed timing stabilization systems using an out-of-loop BOMPD and ATM. The long-term timing fluctuation between the XFEL and the optical laser pulses was successfully reduced to less than 50 fs over 49 h. In recent operation for user experiments, this system has kept a timing fluctuation of ∼50 fs for one week. To evaluate the performance of the timing stabilization systems, we measured the correlation of the long-term simultaneous timing monitoring on two branches of BL3 in 8 h; one is the stational ATM in EH1 and the other is the timing monitor at the sample position for pump–probe experiments in EH2. A linear correlation was obtained with an RMS error of 8.6 fs, which may have originated from mechanical vibrations of the optics and samples, optical pointing fluctuations, and imperfection of the sample flatness. The long-term measurements described in Section 4 were performed by operating both the timing control systems using the out-of-loop BOMPD and the ATM. Since the same result of the timing stability, as shown in Fig. 9 ▸, was obtained without the timing control using the out-of-loop BOMPD, we operate only the timing control using the ATM in recent pump–probe experiments. The timing fluctuation of 50 fs, including the long-term drift, is equivalent to the pulse duration of the optical laser pulse and realizes sufficiently stable measurements of pump–probe experiments at SACLA.
